# 703. Prevalence of Toxigenic and Non-Toxigenic *C. difficile* in Patients Admitted to an Academic-Affiliated Long Term Acute Care Hospital

**DOI:** 10.1093/ofid/ofad500.765

**Published:** 2023-11-27

**Authors:** Amanda F Strudwick, Deepti Suchindran, Sarah Lohsen, Barry Clark, Jeffery D Whatley, Bella Triolo, David Evans, Haley Liakakos, Jack Alperstein, Alex Page, Sarah W Satola, Adhikar Acharya, Lori A Grooms, Ahmed Babiker, Michael H Woodworth

**Affiliations:** Emory University School of Medicine, Atlanta, Georgia; Emory University, Atlanta, Georgia; Emory University School of Medicine, Atlanta, Georgia; Emory University, Atlanta, Georgia; Emory University School of Medicine, Atlanta, Georgia; Emory University, Atlanta, Georgia; Emory University, Atlanta, Georgia; Emory University, Atlanta, Georgia; Emory University, Atlanta, Georgia; Emory University School of Medicine, Atlanta, Georgia; Emory University School of Medicine, Division of Infectious Diseases, Atlanta, Georgia; Emory Healthcare, Decatur, Georgia; Emory Healthcare, Decatur, Georgia; Emory University School of Medicine, Atlanta, Georgia; Emory University, Atlanta, Georgia

## Abstract

**Background:**

*Clostridioides difficile* (C. *difficile*) is the leading hospital associated infection and is highly prevalent among patients admitted to long term care facilities (LTCFs). Both toxigenic and non-toxigenic strains of *C. difficile* colonize patients and produce spores that can persist on healthcare surfaces. Prior trials have shown that administration of non-toxigenic *C. difficile* can reduce recurrent *C. difficile* infection (CDI). We sought to estimate the prevalence of toxigenic and non-toxigenic *C. difficile* in a LTCF as a step toward better understanding if colonization with non-toxigenic *C. difficile* may be protective for CDI.

**Methods:**

Patients admitted to an academic-affiliated long-term acute care facility (or their legally authorized representatives) were approached for verbal consent for collection of peri-rectal, inguinal swabs, and an environmental surface composite swab that were cultured in CCMB-TAL, a differential and selective media for *C. difficile.* Positive cultures underwent nucleic acid extraction and PCR with primers designed to amplify a pan-*C. difficile* target (*rplB*), *C. difficile* toxin (*tcdB*) and the conserved non-coding region carried by non-toxigenic strains instead of the Pathogenicity Locus.

**Results:**

Of 43 occupied LTCF rooms, a total of 32/43 (74.4%) participants were sampled on two floors on one day. The Standard Infection Ratio (SIR) for C.*difficile* in the two quarters prior to sampling were 1.12 and 1.17, at the LTCF. At least one *C. difficile* strain was detected in 8/32 (25%) sampled patients (Fig. 1A). The estimated prevalence of toxigenic *C. difficile* was 5/32 (15.6%). (Fig. 1B, 1D). The estimated prevalence of non-toxigenic *C. difficile* was 5/32 (15.6%) (Fig. 1C, 1D). PCR-based detection of both *tcdB* and the conserved locus in non-toxigenic strains suggested mixed colonization with toxigenic and non-toxigenic strains in 2/32 (6.2%) patients.

**Fig. 1A:**
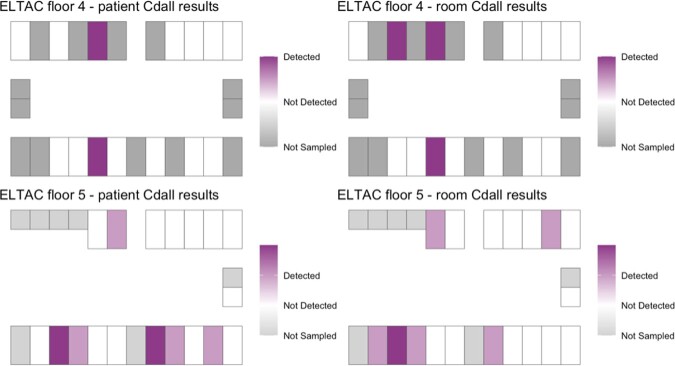
Estimated 25% total prevalence of C.difficile (Cdall: toxigenic and non-toxigenic combined) mapped by patient and room.

**Fig. 1B:**
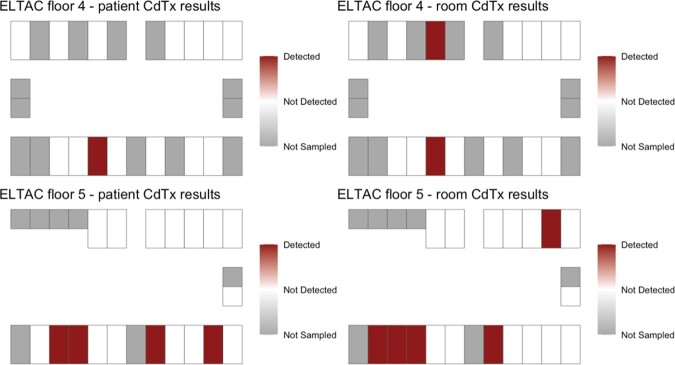
Estimated 16% prevalence of toxigenic C.difficile (CdTx) mapped by patient and room.

**Fig. 1C:**
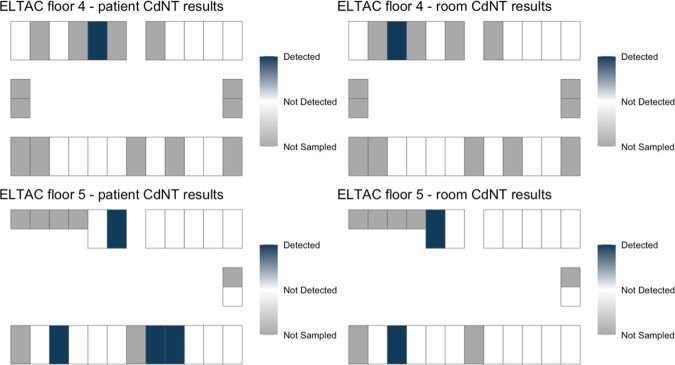
Estimated 16% prevalence of non-toxigenic C. difficile (CdNT) mapped by patient and room.

**Conclusion:**

These data demonstrate that both toxigenic and non-toxigenic strains of *C. difficile* are prevalent in long-term care hospital patients but mixed colonization with both strains is less frequent. Further investigations are needed to determine the relationship between non-toxigenic C. *difficile* prevalence and risk of C. *difficile* infection recurrence.

**Fig. 1D:**
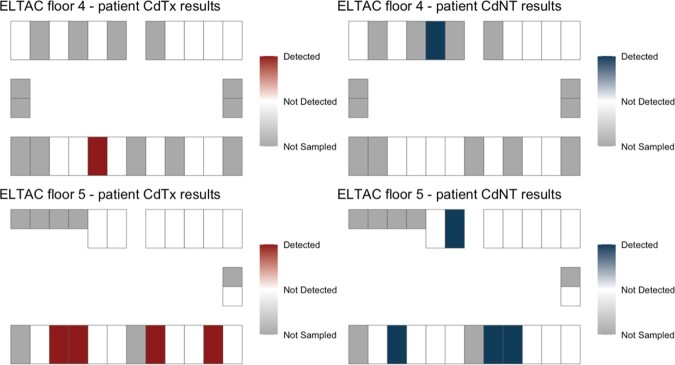
Contrasted patient prevalence of toxigenic (CdTx) vs non-toxigenic (CdNT) C. difficile mapped by patient and room (same data from Figs. 1B & 1C). Co-detection of CdTx and CdNT was seen in 2/32 (6%) patients.

**Disclosures:**

**Ahmed Babiker, MBBS**, Roche: Advisor/Consultant

